# Risk Alleles in/near *ADCY5*, *ADRA2A*, *CDKAL1*, *CDKN2A/B*, *GRB10*, and *TCF7L2* Elevate Plasma Glucose Levels at Birth and in Early Childhood: Results from the FAMILY Study

**DOI:** 10.1371/journal.pone.0152107

**Published:** 2016-04-06

**Authors:** Zahra N. Sohani, Sonia S. Anand, Sebastien Robiou-du-Pont, Katherine M. Morrison, Sarah D. McDonald, Stephanie A. Atkinson, Koon K. Teo, David Meyre

**Affiliations:** 1 Department of Clinical Epidemiology and Biostatistics, McMaster University, Hamilton, Ontario, Canada; 2 Department of Medicine, McMaster University, Hamilton, Ontario, Canada; 3 Department of Pediatrics, Hamilton Health Sciences and McMaster University, Hamilton, Ontario, Canada; 4 Department of Obstetrics and Gynecology, McMaster University, Hamilton, Ontario, Canada; 5 Department of Pathology and Molecular Medicine, McMaster University, Hamilton, Ontario, Canada; Medical University Innsbruck, AUSTRIA

## Abstract

**Background:**

Metabolic abnormalities that lead to type 2 diabetes mellitus begin in early childhood.

**Objectives:**

We investigate whether common genetic variants identified in adults have an effect on glucose in early life.

**Methods:**

610 newborns, 463 mothers, and 366 fathers were included in the present study. Plasma glucose and anthropometric characteristics were collected at birth, 3, and 5 years. After quality assessment, 37 SNPs, which have demonstrated an association with fasting plasma glucose at the genome-wide threshold in adults, were studied. Quantitative trait disequilibrium tests and mixed-effects regressions were conducted to estimate an effect of the SNPs on glucose.

**Results:**

Risk alleles for 6 loci increased glucose levels from birth to 5 years of age (*ADCY5*, *ADRA2A*, *CDKAL1*, *CDKN2A/B*, *GRB10*, and *TCF7L2*, 4.85x10^-3^ ≤ *P* ≤ 4.60x10^-2^). Together, these 6 SNPs increase glucose by 0.05 mmol/L for each risk allele in a genotype score (*P* = 6.33x10^-5^). None of the associations described in the present study have been reported previously in early childhood.

**Conclusion:**

Our data support the notion that a subset of loci contributing to plasma glucose variation in adults has an effect at birth and in early life.

## Introduction

Type 2 diabetes mellitus (T2DM) is a chronic metabolic disorder resulting from the interplay of environmental, genetic, and epigenetic factors [1]. Its prevalence is increasing globally and is forecasted to reach 366 million by the year 2030 [2]. In healthy individuals, plasma glucose is maintained within a narrow physiological range by a balance between insulin secretion and action; glucose tolerance remains constant as long as a reduction in insulin sensitivity at target tissues is compensated by proportionate increases in insulin supply from pancreatic β-cells. Failure of this control mechanism results in dysglycemia and overt T2DM [3], which ultimately have an adverse impact on multiple organ systems.

The incidence of dysglycemia in childhood is increasing [4], a trend that is consistent across ethnic groups [5–8]. Genetic factors contribute to this disorder. Heritability estimates for fasting plasma glucose (FPG) range between 38% and 51% [9,10]. Genome wide association studies (GWAS) to date have identified 39 single nucleotide polymorphisms (SNPs) that lead to increased FPG levels in adults [11–13]. Similarly, studies in children and adolescents have reported SNPs related to variation in glucose. Notably, Kelliny *et al* and Weedon *et al* [14,15] found associations between genetic variants in *MTNR1B*, *GCK*, *G6PC2*, and *SLC30A8* and FPG in children and adolescents from the European Youth Heart Study (EYHS). Additionally, a meta-analysis of GWAS SNPs in 6,000 adolescents aged 9–16 years found 16 variants associated with FPG [16]. A majority of these SNPs increase FPG by small increments, specifically 0.02–0.06 mmol/L per risk allele. To date, no systematic investigation of the contribution of genetic variants on plasma glucose at birth and early life has taken place.

The specific aim of this paper is to investigate the contribution of genetic variants, analyzed separately and together as a genotype score, on plasma glucose in children from birth to 5 years of age.

## Methods

### Participants

Data for the present study were acquired from the FAMILY (Family Atherosclerosis Monitoring In earLY life) cohort. Details on FAMILY have been previously described [17]. Briefly, 901 newborns (with their parents and siblings), recruited from the greater Hamilton areas, were prospectively followed for up to 7 years, with a planned follow-up of at least 10 years. Biochemical and anthropometric measurements were collected for all participants at birth and during follow-up. The primary objective of the FAMILY study was to evaluate the early determinants of cardio-metabolic complications in children.

For the present study, we aimed to investigate the effect of genetic variants on glucose in early life and thus included newborns from the FAMILY study in whom serial glucose measurements, starting at birth, were available. Plasma glucose measurements were available at birth, 3, and 5 years of age. Glucose measurements at birth were taken from cord blood and participants were fasting at 3 and 5 years. The samples were stored frozen and were later analyzed.

Coordination site of the FAMILY study took place at the Population Health Research Institute, Hamilton Health Sciences and McMaster University, Hamilton, ON, Canada. Written informed consent was obtained from all participants and all experiments were performed in accordance with relevant guidelines and regulations. Ethics boards at Hamilton Health Sciences, St Joseph’s Health Center, and Joseph Brant Memorial Hospital in Hamilton and Burlington, Ontario, Canada approved the FAMILY study [17].

### DNA extraction, SNP selection, and genotyping

DNA was extracted from buffy coats and genotyping was conducted using the Illumina Cardio-Metabochip (San Diego, CA, USA). SNPs significantly associated with FPG from GWA-studies at *P* < 5x10^-8^ were tested in the present analysis [11,12,18,19]. Based on this criterion, 39 SNPs were selected. Thirty-seven of the 39 SNPs or their proxies (SNPs in linkage disequilibrium at *r*^2^ > 0.85) were available on the Cardio-Metabochip array. Proxy SNPs were identified using 1000 Genomes data *via* the SNAP software. *VPS13C* rs4502156 was used as a proxy for rs7173964 (*r*^2^ = 0.97) and *MADD* rs11033182 was used as proxy for rs7944584 (*r*^2^ = 0.88). Data for *G6PC2* rs560887 and *GLIS3* rs7034200 were not available. All 37 SNPs were independent from each other (*r*^2^ < 0.2 in European population data of the 1000 Genomes Project). A list of the included SNPs is presented in S1 Table.

Standard assessments were conducted to ensure quality of genotyping. All SNPs were in agreement with Hardy Weinberg Equilibrium (*P* > 0.001) and displayed call rates greater than 95% (S1 Table). After quality assessment, the following exclusions were made: 5 families were excluded because of recurrent inconsistencies in Mendelian transmission patterns, 5 fathers, 1 mother, and 3 children were removed for discrepancies between the reported and genetically determined sex, and 4 children were excluded due to cryptic relatedness. After quality control, 610 newborns, 463 mothers, and 366 fathers were included in the analysis.

### Statistical methods

Statistical analyses were performed using PLINK (http://pngu.mgh.harvard.edu/~purcell/plink/), R (version 3.0.2), and UNPHASED (version 3.1.7). For each of the 37 SNPs, the glucose-elevating allele previously identified in adults was used as the risk allele. Genotypes were coded as 0, 1, and 2 designating the copies of the risk allele. An overall genotype score was calculated for each individual by allele counting, and thus the score could range from 0 to 74. We used an unweighted genotype score as recommended by Janssens *et al*. [20]. We created a second genotype score of only SNPs significantly associated with glucose in early childhood to estimate the estimate the cumulative contribution of these genes. Individuals with more than 10% missing genotypes were excluded from the analysis. Missing values for remaining individuals in the genotype score were imputed using the arithmetic average of the coded genotypes. Siblings of “index” children (n = 33) were excluded from the regression analysis due to familial relatedness.

Effect of the SNPs on plasma glucose in children was estimated using two methods. First, family-based tests, described by F. Dudbridge [21], were performed at each time point (i.e. birth, 3 year, and 5 year) using UNPHASED. Traditionally, family-based tests, such as Quantitative trait disequilibrium test (QTDT), assess the existence of transmission of alleles in excess of expected proportions in pedigrees and offer advantages over classical regression analysis as they are not as susceptible to non-normality and eliminate potential for confounding from population stratification since parents act as controls for their ‘affected’ offspring [22]. Using the method described by Dudbridge, we used complete and incomplete trios to perform the test. SNPs that were significant at multiple time points were compared using a Z-test or one-way ANOVA. The second method used was mixed-effect linear regression. Mixed-effects models use data for each individual at various time points to present an estimate of the overall mean annual change in plasma glucose for each SNP tested. A random intercept was allocated to each child. Each SNP and genotype score was tested independently and the model was adjusted for sex, age, birth weight, and population substructure using the first 10 axes of variation from Principal Components Analysis. PCA was conducted using EIGENSTRAT. Bonferroni corrected P values are routinely applied to exploratory genetic association studies. However, they are over conservative given the high prior likelihood of association in post-GWAS experiments. In agreement with other post-GWAS experiments [14,16], we considered a *P* < 0.05 as statistically significant. Two-sided *P* values are reported for all statistical tests.

## Results

Summary characteristics for included children, mothers, and fathers are presented in Table 1. The mean age for mothers and fathers in this cohort was 32.8 years and 33.9 years, respectively. Mean glucose level at birth in children was 4.28 mmol/L. On average, children had a gestational age of 39 months and a birth weight of 3.41 kg. A majority of individuals in the FAMILY study were of European descent (92.8% mothers; 89.3% fathers; 91.1% offspring).

**Table 1:** Phenotype characteristics at baseline of the participants involved in the present study.

Characteristic	Child (n = 610)	Mother (n = 463)	Father (n = 366)
	Mean (SD) / %	Mean (SD) / %	Mean (SD) / %
Age (years)	NA	32.8 (4.67)	33.9 (5.65)
Baseline glucose (mmol/L)	4.28 (0.97)	4.46 (0.68)	5.08 (0.79)
Birth weight (kg)	3.41 (0.63)	NA	NA
Gestational age (months)	39.0 (2.00)	NA	NA
Current smokers	NA	12%	NA
Previous T2DM or GDM	NA	7%	NA
Female	51%	100%	0%

T2DM: Type 2 diabetes mellitus; GDM: Gestational diabetes mellitus; NA: not applicable/available.

### Genetic variants associated with glucose

#### Results from family-based association tests

Quantitative trait disequilibrium tests were performed at birth, 3 years, and 5 years (Table 2). The rs6943153 SNP near *GRB10* was associated with glucose at all three time-points in a directionally consistent manner (birth: β = 0.07, *P* = 4.86x10^-3^; 3 years: β = 0.05, *P* = 7.16x10^-3^; 5 years: β = 0.03, *P* = 1.75x10^-2^) and the effect size between time-points did not differ (*P* = 0.78). A second SNP, *CDKN2A/B* rs10811661, showed a significant and directionally consistent association with glucose at birth and 3 years (birth: β = 0.11, *P* = 1.24x10^-2^; 3 years: β = 0.12, *P* = 4.85x10^-3^) and a trend toward association at 5 years (*P* = 0.09). The effect was not statistically different between time-points (*P* = 0.89). Lastly, risk allele for rs576674 near *KL* led to a higher glucose level at 5 years (5 years: β = 0.10, *P* = 1.92x10^-2^), but no association for this SNP was observed at birth or at 3 years.

**Table 2:** SNPs associated with plasma glucose in children using quantitative trait disequilibrium test.

Gene	SNP	Risk allele	Time point	Effect (SE) (mmol/L)	*P* value
*CDKN2A/B*	rs10811661	T	Birth	0.11 (0.056)	1.24 x 10^−2^
*GRB10*	rs6943153	T	Birth	0.07 (0.046)	4.86 x 10^−3^
*CDKN2A/B*	rs10811661	T	3 years	0.12 (0.049)	4.85 x 10^−3^
*GRB10*	rs6943153	T	3 years	0.05 (0.039)	7.16 x 10^−3^
*KL*	rs576674	G	5 years	0.10 (0.042)	1.92 x 10^−2^
*GRB10*	rs6943153	T	5 years	0.03 (0.035)	1.75 x 10^−2^

#### Results from mixed-effect regression analyses

Mixed-effects regression models were used to test the global effects of SNPs on plasma glucose from birth to the age of 5 years. Of the 37 SNPs tested, *CDKAL1* rs9368222 (β = 0.08, *P* = 1.22x10^-2^), *ADCY5* rs11708067 (β = 0.07, *P* = 4.07x10^-2^), *ADRA2A* rs10885122 (β = 0.09, *P* = 2.35x10^-2^), and *TCF7L2* rs4506565 (β = 0.06, *P* = 4.60x10^-2^) were associated with glucose levels in children (Table 3). All significant results were directionally consistent with the initial GWAS reports in adult populations. Results for all SNPs tested are presented in S2 Table.

**Table 3:** SNPs associated with plasma glucose in children using mixed-effects regression.

Gene	SNP	Risk allele	Effect (SE) (mmol/L)	*P* value
*ADCY5*	rs11708067	A	0.07 (0.034)	4.07 x 10^−2^
*CDKAL1*	rs9368222	A	0.08 (0.030)	1.22 x 10^−2^
*TCF7L2*	rs7903146	T	0.06 (0.029)	4.60 x 10^−2^
*ADRA2A*	rs10885122	G	0.09 (0.040)	2.35 x 10^−2^

### Genotype score

First, we tested the association of an unweighted genotype score of all 37 SNPs with children’s glucose levels, using mixed-effects regression models. The unweighted genotype score of all 37 variants had a mean of 36.75 (SD = 3.83) in offspring and raised plasma glucose by 0.008 mmol/L for each unit increase in the score. However, the genotype score was not significantly associated with glucose levels (*P* = 0.10). To estimate the cumulative effect of significantly associated SNPs on glucose, we created a second genotype score of SNPs that were convincingly associated with glucose in our primary analysis (*TCF7L2*, *ADCY5*, *CDKAL1*, *ADRA2A*, *CDKN2A/B*, and *GRB10*). *KL* was not included in our genotype score as this signal was likely a false positive finding (see Discussion). The 6 SNPs, significant in our primary analysis, cumulatively increased plasma glucose levels by 0.053 mmol/L per risk allele (*P* = 6.33x10^-5^).

## Discussion

Using a longitudinal study of newborns and their families, we report 6 SNPs in or near *TCF7L2*, *ADCY5*, *CDKAL1*, *ADRA2A*, *CDKN2A/B*, and *GRB10* that modulate plasma glucose in early life. Together, these 6 SNPs increase glucose by 0.05 mmol/L for each risk allele in a genotype score. *KL* was associated with glucose at 5 years, using QTDT, but did not have an effect at birth or 3 years of age. None of the associations described in the present study have been reported previously in early childhood.

The FAMILY study provides a unique opportunity to investigate the effects of genetic variants on metabolic traits at birth and in the early years of life. This cohort is among a few worldwide to longitudinally follow children from birth [17]. The results from this study suggest that a subset of genes, which modulate adult glucose levels, have already an impact in early childhood. From among these, *CDKAL1* and *ADCY5* also contribute to variations in birth weight [23,24], pancreatic beta-cell function/insulin secretion [25–27], and increase risk for T2DM [13]. Since these genes influence parameters of growth *in utero*, they likely have an impact on glucose in fetal life [28]. Conversely, *TCF7L2*, *ADRA2A*, *CDKN2A/B*, and *GRB10* may start to influence glucose levels in the post-natal period. The statistically significant association of glucose with a SNP on *KL* is likely a false positive since it was only apparent at one time point.

A meta-analysis of the ALSPAC, GENDAI, obese French cases, French controls, EYHS, and Raine cohorts of various Europeans and Australian descents identified several SNPs associated with fasting glucose in 6,000 adolescents between the ages of 9 and 16 [16]. Only *ADCY5* was significantly associated with glucose in both our study and the meta-analysis. An additional 2 genes, *TCF7L2* and *ADRA2A*, were tested, and while significant in our analysis, were found to not be associated with glucose in this meta-analysis: *TCF7L2* -0.0088 (-0.030, 0.012) and *ADRA2A* 0.0076 (-0.016, 0.031). The lack of consistency between our findings and those from this meta-analysis may result from population stratification. Our analyses were adjusted for the first 10 principle components to account for any population substructure present in our cohort. By contrast, Barker *et al* adjusted for age, sex, BMI but not population stratification for most cohorts, with the exception of EYHS and Raine for which country and principle components were used, respectively [16]. A meta-regression with an indicator of population origin as a covariate was not performed. As the cohorts differed in ethnic origin and selection, heterogeneity may be present in the meta-analysis leading to large variances and subsequent statistically insignificant findings for the genes we found to be associated with glucose [29]. For genes associated with glucose in the meta-analysis and another study of adolescents by Kelliny *et al* [14] that were not significant in our cohort, notably *MTNR1B*, *GCK*, and *SLC30A8*, it is likely that we were underpowered to detect these effects.

Our study has several strengths. Most importantly, ours is the first report investigating SNPs that affect glucose levels at birth and in early childhood. Second, a family-based design allowed us to test the association of SNPs with glucose using both classical regression analyses as well as family-based association tests. The benefit of using both methods was to circumvent pitfalls unique to either method. Third, the longitudinal nature of the data facilitated use of mixed-effects models, which robustly report overall association of the SNP with the outcome after considering multiple time points. Lastly, investigation of an up-to-date list of 37 SNPs allows us to report the most current state of evidence.

This study also has several limitations. First, our study had a relatively modest sample size, which adversely affected our power to detect associations with small effect sizes and low risk allele frequencies. Second, our analysis also had fewer fathers than mothers. Unfortunately, this is a common pitfall of family-based designs where mothers often bring children to clinic visits and thus are included more easily than fathers. Thirdly, we used cord blood measurements at birth since fasting glucose samples from newborns are not available. Use of cord blood may have biased our analysis since the phenotype is not defined in the same way at all-time points. Nonetheless, cord blood glucose is frequently used in newborn studies [30].

Future studies investigating the genetic determinants of glucose traits should undertake an agnostic search for SNPs associated with glucose levels in early childhood in order to i) confirm findings reported in this study, ii) resolve inconsistencies in the present literature, and iii) capture novel polymorphisms that have an effect on plasma glucose only in early life. Additionally, future studies would benefit from exploring both regression methods as well as family based tests in order to maximize detection of relevant genes.

In conclusion, we report the effects of SNPs in or near *ADCY5*, *ADRA2A*, *CDKAL1*, *CDKN2A/B*, *GRB10*, and *TCF7L2* on plasma glucose in early childhood. Our findings help elucidate the pathology of dysglycemia, specifically its development as a result of changes in early life. These results may ultimately contribute to early prevention of T2DM and its complications.

## Supporting Information

S1 Table Risk allele frequencies, call rates, and p-values for the Hardy Weinberg Equilibrium test for all genes tested. (DOCX)

S2 Table Results from mixed effects regression analysis in children. (DOCX)
