# Supplementary material for: Risk Alleles in/near ADCY5, ADRA2A, CDKAL1, CDKN2A/B, GRB10, and TCF7L2 Elevate Plasma Glucose Levels at Birth and in Early Childhood: Results from the FAMILY Study
Source: PLoS One. 2016 Apr 6;11(4):e0152107. doi: 10.1371/journal.pone.0152107 (PMC4822946; doi:10.1371/journal.pone.0152107)
Supplement: S1 Table — (DOCX) [file pone.0152107.s001.docx]

**S1 Table 1** Risk allele frequencies, call rates, and *P*-values for the Hardy Weinberg Equilibrium test for all genes tested

| Gene | SNP | HWE P value | Risk Allele | RAF | Call Rates |
| --- | --- | --- | --- | --- | --- |
| *P2RX2* | rs10747083 | 0.8446 | A | 0.70 | 96.13948 |
| *CDKN2B* | rs10811661 | 0.03223 | T | 0.84 | 99.19054 |
| *MTNR1B* | rs10830963 | 0.03473 | G | 0.28 | 98.94147 |
| *ADRA2A* | rs10885122 | 1 | G | 0.87 | 98.94147 |
| *CENTD2* | rs11603334 | 1 | G | 0.87 | 99.12827 |
| *CRY2* | rs11605924 | 0.5164 | A | 0.24 | 98.75467 |
| *PDX1* | rs11619319 | 0.5046 | G | 0.24 | 99.4396 |
| *ADCY5* | rs11708067 | 0.1406 | A | 0.77 | 98.63014 |
| *AMT* | rs11715915 | 0.2844 | C | 0.71 | 99.12827 |
| *SLC2A2* | rs11924648 | 0.8631 | A | 0.86 | 99.00374 |
| *C2CD4A/B* | rs12440695 | 0.438 | A | 0.63 | 99.19054 |
| *GCKR* | rs1260326 | 0.5626 | T | 0.42 | 98.94147 |
| *SLC30A8* | rs13266634 | 0.02962 | C | 0.71 | 99.12827 |
| *DPYSL5* | rs1371614 | 0.7397 | T | 0.24 | 99.31507 |
| *OR4S1* | rs1483121 | 0.8685 | G | 0.86 | 99.31507 |
| *FADS1* | rs174550 | 0.206 | T | 0.66 | 98.8792 |
| *RREB1* | rs17762454 | 0.4023 | T | 0.26 | 99.31507 |
| *DGKB-TMEM195* | rs2191349 | 0.8706 | T | 0.55 | 99.19054 |
| *GIPR* | rs2302593 | 0.5194 | C | 0.50 | 99.19054 |
| *GLS2* | rs2657879 | 0.5653 | G | 0.17 | 98.06974 |
| *PROX1* | rs340874 | 1 | C | 0.56 | 99.2528 |
| *WARS* | rs3783347 | 1 | G | 0.81 | 99.19054 |
| *DNLZ* | rs3829109 | 0.7672 | G | 0.71 | 98.6924 |
| *VPS13C* | rs4502156 | 0.8698 | T | 0.56 | 98.8792 |
| *TCF7L2* | rs4506565 | 1 | T | 0.32 | 99.2528 |
| *PCSK1* | rs4869272 | 0.5632 | T | 0.70 | 99.19054 |
| *KL* | rs576674 | 0.06749 | G | 0.19 | 98.8792 |
| *TOP1* | rs6072275 | 1 | A | 0.16 | 99.2528 |
| *FOXA2* | rs6113722 | 1 | G | 0.96 | 98.81694 |
| *GRB10* | rs6943153 | 0.928 | T | 0.34 | 98.19427 |
| *GCK* | rs6975024 | 0.6579 | C | 0.16 | 98.6924 |
| *IGF2BP2* | rs7651090 | 0.1264 | G | 0.30 | 99.00374 |
| *ZBED3* | rs7708285 | 0.6194 | G | 0.28 | 99.00374 |
| *CDKAL1* | rs9368222 | 0.1226 | A | 0.27 | 99.4396 |
| *PPP1R3B* | rs983309 | 1 | T | 0.11 | 98.94147 |
| *PPP1R3B* | rs9987289 | 0.7987 | A | 0.09 | 99.31507 |
| *MADD* | rs11039182 | 0.4772 | G | 0.28 | 96.82441 |
