# Supplementary material for: Risk Alleles in/near ADCY5, ADRA2A, CDKAL1, CDKN2A/B, GRB10, and TCF7L2 Elevate Plasma Glucose Levels at Birth and in Early Childhood: Results from the FAMILY Study
Source: PLoS One. 2016 Apr 6;11(4):e0152107. doi: 10.1371/journal.pone.0152107 (PMC4822946; doi:10.1371/journal.pone.0152107)
Supplement: S2 Table — (DOCX) [file pone.0152107.s002.docx]

**S2 Table 2** Results from mixed effects regression analysis in children.

| Gene | SNP | Beta | SE | P |
| --- | --- | --- | --- | --- |
| *P2RX2* | rs10747083 | 0.009396437 | 0.031071559 | 0.762337394 |
| *CDKN2B* | rs10811661 | 0.009127848 | 0.039079794 | 0.815319211 |
| *MTNR1B* | rs10830963 | -0.056401664 | 0.029289801 | 0.054149123 |
| *ADRA2A* | rs10885122 | 0.090598483 | 0.039988383 | 0.023474655 |
| *MADD* | rs11039182 | -0.043937502 | 0.030222033 | 0.145995246 |
| *CENTD2* | rs11603334 | -0.06071227 | 0.042279229 | 0.151007075 |
| *CRY2* | rs11605924 | -0.007641092 | 0.02751849 | 0.781264767 |
| *PDX1* | rs11619319 | 0.034559988 | 0.032263751 | 0.284092638 |
| *ADCY5* | rs11708067 | 0.070406878 | 0.034404577 | 0.040713169 |
| *AMT* | rs11715915 | 0.018144711 | 0.029277267 | 0.535419579 |
| *SLC2A2* | rs11924648 | 0.059563975 | 0.039882348 | 0.135308398 |
| *C2CD4A/B* | rs12440695 | -0.034039017 | 0.029297582 | 0.245301644 |
| *GCKR* | rs1260326 | 0.026568658 | 0.02840498 | 0.349606871 |
| *SLC30A8* | rs13266634 | -0.049892328 | 0.031795934 | 0.116614904 |
| *DPYSL5* | rs1371614 | -0.015904887 | 0.032151101 | 0.620817668 |
| *OR4S1* | rs1483121 | -0.00201858 | 0.039439573 | 0.959180823 |
| *FADS1* | rs174550 | -0.014155513 | 0.028772276 | 0.622730321 |
| *RREB1* | rs17762454 | 0.002217976 | 0.03095845 | 0.942885522 |
| *DGKB-TMEM195* | rs2191349 | 0.012645199 | 0.027709811 | 0.648142811 |
| *GIPR* | rs2302593 | -0.015444829 | 0.02855476 | 0.588587146 |
| *GLS2* | rs2657879 | 0.011018043 | 0.037532176 | 0.769092159 |
| *PROX1* | rs340874 | 0.023039986 | 0.027465558 | 0.401543214 |
| *WARS* | rs3783347 | -0.017437792 | 0.034381898 | 0.612028919 |
| *DNLZ* | rs3829109 | 0.019792842 | 0.030891813 | 0.521707995 |
| *VPS13C* | rs4502156 | -0.006007607 | 0.028382696 | 0.832368858 |
| *TCF7L2* | rs4506565 | 0.058462028 | 0.029294429 | 0.045969795 |
| *PCSK1* | rs4869272 | 0.048320375 | 0.029966093 | 0.106852814 |
| *KL* | rs576674 | -0.041240671 | 0.034932826 | 0.23777331 |
| *TOP1* | rs6072275 | -0.042540622 | 0.037980015 | 0.262680046 |
| *FOXA2* | rs6113722 | 0.096148391 | 0.070464416 | 0.172411613 |
| *GRB10* | rs6943153 | 0.019652059 | 0.029258483 | 0.501793499 |
| *GCK* | rs6975024 | 0.017843959 | 0.038032401 | 0.638942543 |
| *IGF2BP2* | rs7651090 | 0.037634511 | 0.029519292 | 0.202340245 |
| *ZBED3* | rs7708285 | 0.023282106 | 0.031052614 | 0.453397363 |
| *CDKAL1* | rs9368222 | 0.075086352 | 0.029966739 | 0.012222433 |
| *PPP1R3B* | rs983309 | 0.060848235 | 0.046196066 | 0.187780474 |
| *PPP1R3B* | rs9987289 | 0.056575695 | 0.052218676 | 0.278614079 |
